# The effects of assisted reproduction technologies on metabolic health and disease^†^

**DOI:** 10.1093/biolre/ioaa224

**Published:** 2020-12-16

**Authors:** Maria Florencia Heber, Grażyna Ewa Ptak

**Affiliations:** 1 Malopolska Centre of Biotechnology, Jagiellonian University, Krakow, Poland; 2 Faculty of Biosciences, University of Teramo, Teramo, Italy

**Keywords:** metabolic syndrome, assisted reproductive technologies, epigenetics, cardiovascular disease, type 2 diabetes

## Abstract

The increasing prevalence of metabolic diseases places a substantial burden on human health throughout the world. It is believed that predisposition to metabolic disease starts early in life, a period of great susceptibility to epigenetic reprogramming due to environmental insults. Assisted reproductive technologies (ART), i.e., treatments for infertility, may affect embryo development, resulting in multiple adverse health outcomes in postnatal life. The most frequently observed alteration in ART pregnancies is impaired placental nutrient transfer. Moreover, consequent intrauterine growth restriction and low birth weight followed by catch-up growth can all predict future obesity, insulin resistance, and chronic metabolic diseases. In this review, we have focused on evidence of adverse metabolic alterations associated with ART, which can contribute to the development of chronic adult-onset diseases, such as metabolic syndrome, type 2 diabetes, and cardiovascular disease. Due to high phenotypic plasticity, ART pregnancies can produce both offspring with adverse health outcomes, as well as healthy individuals. We further discuss the sex-specific and age-dependent metabolic alterations reflected in ART offspring, and how the degree of interference of a given ART procedure (from mild to more severe manipulation of the egg) affects the occurrence and degree of offspring alterations. Over the last few years, studies have reported signs of cardiometabolic alterations in ART offspring that are detectable at a young age but that do not appear to constitute a high risk of disease and morbidity per se. These abnormal phenotypes could be early indicators of the development of chronic diseases, including metabolic syndrome, in adulthood. The early detection of metabolic alterations could contribute to preventing the onset of disease in adulthood. Such early interventions may counteract the risk factors and improve the long-term health of the individual.

## Introduction

In the past few decades, the incidence of metabolic diseases and metabolic syndrome in particular has increased dramatically in Western countries, becoming a global epidemic. Metabolic syndrome is a cluster of metabolic alterations characterized by obesity, insulin resistance, hypertension, altered glucose metabolism, and dyslipidaemia [1]. It is associated with increased risk of cardiovascular disease (CVD) and type 2 diabetes (T2D), among others [2]. The combination of at least three metabolic alterations forms the basis for metabolic syndrome diagnosis, whereas the presence of at least one alteration increases the risk of developing the syndrome later in life and furthermore represents a high risk of CVD [3]. Although metabolic syndrome in adulthood is undoubtedly caused by multiple factors, including modifiable lifestyle, fetal life may represent a critical window in which individuals are predisposed to metabolic syndrome later in life.

A strong relationship between adverse fetal environment and the onset of metabolic alterations has been extensively reported (reviewed in [4–6]). Human and animal studies have shown that both maternal and paternal undernutrition, obesity, stress, and exposure to endocrine disruptors can all induce metabolic alterations, leading to increased risk of T2D, CVD, and metabolic syndrome development.

The developmental origins of health and disease (DOHaD) hypothesis states that any insult during critical times of development (in utero or early life) has the ability to modify the individual’s phenotype, which can lead to the onset of diseases during later life [7, 8]. This phenomenon, called developmental programming [9, 10], states that the influence of different factors—such as lifestyle, diet, environmental pollutants, medical and pharmaceutical interventions, and hormones, among others—during periods of phenotypic plasticity can affect the organism by altering its development [11]. Though a variety of different insults are capable of programming fetal development, there is great similarity in the phenotypic outcome. The majority of these insults result in placental alterations, which may lead to intrauterine growth restriction (IUGR) and catch-up growth, which in later life, predisposes the offspring to adult disease [4].

Another example of an environmental insult is the use of assisted reproductive technologies (ART), on which we focus in this review. ART include various procedures of assisted conception used in cases of female or male infertility, and they have increased dramatically over the years. These procedures include various steps, including ovarian stimulation, oocyte/sperm recovery, gamete and embryo manipulation, cryopreservation, in vitro culture, and embryo transfer [12]. The degree of interference of each procedure also varies, and include exclusively hormonal treatment of the patient, or in vitro procedures*:* from mild interventions on gametes, like in vitro fertilization (IVF) to more severe interference as IntraCytoplasmic Sperm Injection (ICSI), or to the embryo, like trophectoderm or blastomere biopsy (BB). These steps are known to take place at critical times of development, when the genome is undergoing significant epigenetic remodeling and is vulnerable to environmental factors, and, if disturbed, could adversely influence developmental programming.

Little is known about the causes of developmental defects in ART-derived embryos occurring during early pregnancy, as most of them are detrimental and therefore, only the most robust and healthy embryos are able to develop to term. ART-derived neonates usually look healthy, though placental alterations of different degree and severity are frequently observed both in humans and in animals [13–17]. The authors’ studies using sheep as a model showed defective placental vascularization and nutrient transport in early pregnancies obtained by ART [18, 19]. Temporal observations of ART-derived sheep embryos during early placentation demonstrated that their underdeveloped cardiovascular system was the main cause of embryonic growth retardation and death [18]. These previous studies indicated that ART embryos with major defects are eliminated during placental vessel development. Placental vasculogenesis may therefore constitute a developmental bottleneck for ART embryos—in other words, a developmental life-or-death decision stage. High mortality of embryos with cardiovascular defects, could explain why only minor or nondetectable alterations (if any) are present at birth following ART pregnancies.

Even if a neonate looks healthy, the underdeveloped placental vascular network in ART pregnancies (caused by triggering less efficient catabolic removal and nutrient provision in fetal tissues) may cause developmental adaptations and may contribute to long-term health consequences. The placenta has a strong impact not only on fetal heart development but also on the offspring’s future adulthood because it is a programming agent for cardiometabolic diseases. Increased blood pressure in ART-derived individuals [20, 21] could be the result of an underdeveloped vascular system. Altogether, the application of ART may induce impaired vasculogenesis early in gestation. This impairment may lead, in more severe circumstances, to embryo growth arrest and, in less severe ones, to fetal growth restriction throughout the pregnancy, programming the offspring to be susceptible to cardiometabolic disease.

Although the great majority of children born through ART are healthy, its use is associated with several adverse health outcomes, e.g., risk of gestational diabetes [22], pregnancy hypertension, and altered placental development and function [13, 23–25], as well as adverse perinatal outcomes, e.g., perinatal mortality, congenital defects, and epigenetic disorders [23, 26]. The most frequently observed ART complications include: (i) abnormalities and defects in the placenta (e.g., defects in nutrient transport, morphological abnormalities, impaired vasculogenesis, antioxidant defects) [14–17, 23]; (ii) low birth weight (human: [27, 28], rodents: [15, 16]; and (iii) IUGR [29–32]. IUGR and low birth weight, followed by catch-up growth has been associated with detrimental, long-term metabolic consequences and is known to predict obesity, insulin resistance, and CVD in adults [33, 34]. Thus, the adverse outcomes of ART during perinatal life have the potential to result in adverse metabolic health in postnatal life. Even if the influence of ART on a detrimental metabolic phenotype is still not completely clear, it has been suggested that children born following ART might be at higher risk of cardiovascular and metabolic alterations [25, 35].

## ART and metabolic alterations

### Evidence from human studies

**Figure 1:**
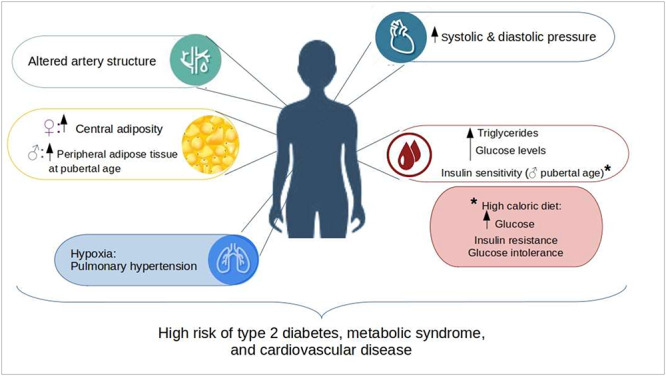
Evidence of cardiometabolic alterations from human studies. The illustration represents the main metabolic alterations found in ART-conceived children in the main organ systems involved in the pathogenesis of metabolic syndrome. The presence of at least one of the alterations represented in the figure increases the risk that these children will develop type 2 diabetes, metabolic syndrome, and cardiovascular disease later in life.

Over the last several years, epidemiological studies have focused on the long-term metabolic health of ART-conceived children (see Figure 1). Studies have reported that young children conceived by both IVF and ICSI (the two most common ART) presented high systemic and diastolic blood pressure [20, 21, 36, 37]. Other authors have found increased blood pressure only in children conceived using hormonal induction of ovulation following ICSI or IVF [38]. Moreover, Scherrer et al. reported that ART-conceived children presented differences in systemic circulation, artery structure, and observed pulmonary hypertension in hypoxic conditions [39]. Along with this study, Chen et al. reported that IVF-conceived children, when challenged with a high-caloric diet, have increased blood pressure [36]. Overall, these alterations in the cardiovascular system could result in a high risk of cardiometabolic disease, which increases under stress situations.

Other signs of metabolic dysregulations have been observed in young children and adolescents conceived through ART. High fasting glucose levels have been reported in young ART-conceived children [21, 38]. In particular, Ceelen et al. found high glucose levels in pubertal IVF children independently of early life factors (e.g., birth weight and gestational age) or parental characteristics (e.g., parental age, subfertility, and body weight) [21]. Chen et al. noted only reduced peripheral insulin sensitivity in young adults conceived by IVF. When this cohort was challenged with a high-caloric diet, they presented high fasting glucose levels, glucose intolerance, and insulin resistance [36]. However, other studies have not found any alterations on glucose metabolism after ART [36, 37, 40]. Other studies have also reported increased peripheral adipose tissue in ART-conceived children [20, 21, 41]. Moreover, Belva et al. reported an increase in central adipose tissue in females conceived by ICSI, whereas young males showed an increase in peripheral adiposity when they reached advanced pubertal age [42]. In particular, Ceelen et al. observed a trend of increased, but not significant, body fat composition in late childhood; however at pubertal age, this high-fat deposition becomes more evident [41]. A few studies have also noted high levels of triglycerides [37]. Taken together, these studies show that ART could influence lipid and glucose metabolism in ART-conceived children, resulting in a high risk of T2D and metabolic syndrome. Moreover, the observed alterations seem to be more evident at pubertal age rather than in early childhood, suggesting that even if ART-conceived children seem healthy at a young age, is necessary to continue with follow-up studies, since metabolic alterations could arise later in life and influence the development of adult-onset disease.

### Evidence from animal studies

**Figure 2:**
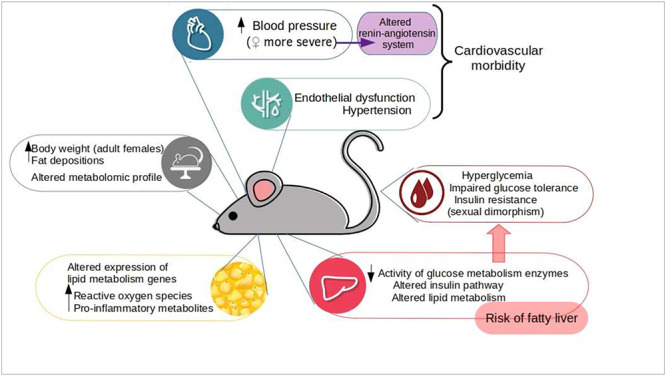
Evidence of cardiometabolic alternations from animal studies. The illustration summarizes the main metabolic alterations reported in ART-produced offspring from animal models and the possible molecular mechanisms behind the alterations found. Animal studies support the evidence of ART-induced alterations in human studies and provide evidence of alterations in the cardiovascular system, adipose tissue, and liver, which are the main axes involved in the development of metabolic syndrome.

Evidence from human studies are controversial because most ART-conceived children are still young and because it is problematic to elucidate whether the adverse outcomes are related to the procedures per se or to parental factors. Animal models have been advantageous, since you can “remove” the underlying parental infertility, as well as other confounding factors (e.g., ethnicity, genetics) and focus on the outcomes specifically correlated to the ART procedures. Alterations found in ART-conceived children have been confirmed and supported by animal models (see Figure 2). Several studies have demonstrated that mice conceived by various ART exhibit cardiovascular and metabolic abnormalities. Watkins et al. reported that embryo culture is able to induce increased blood pressure in mouse offspring, with more severe effects on female offspring, indicative of a risk of hypertension [43]. Other groups have found endothelial dysfunction and hypertension in IVF-produced mice offspring [44, 45], which could be associated with risk of premature cardiovascular morbidity. Moreover, ART-produced female offspring showed altered expression of the renin-angiotensin system, which regulates blood pressure and is known to play a role in the pathogenesis of CVD [46]. Several experimental studies in mouse have demonstrated that ART can alter glucose metabolism, from simple hyperglycaemia to impaired glucose tolerance, which can lead to hyperinsulinemia and in some cases, to insulin resistance [36, 47–49]. Due to sex-related developmental alterations observed in ART offspring, these results are apparently conflicting. Some studies have shown that female mouse offspring obtained by IVF or ICSI or under suboptimal embryo culture conditions displayed glucose intolerance and high glucose levels, whereas male offspring presented normal glucose homeostasis [47, 50]. Conversely, our studies showed that BB (an essential technique for performing preimplantation genetic diagnosis (PGD), a screening test that can detect genetic abnormalities of embryos before their transfer in utero) increased the body weight of the resulting male offspring as early as in the second week of life [51]. There is limited understanding of the postnatal consequences and safety of BB procedure; however, it cannot be excluded that such an invasive ART procedure affects the long-term postnatal developmental programming of male mice, suggesting that PGD could be a risk factor for late-onset, metabolic disease predisposition. Other studies have shown that male mouse offspring are more susceptible to displaying abnormal glucose metabolism [48, 52]. Sexual dimorphism has also been observed in other metabolic alterations. Feuer et al. reported that female IVF-produced offspring had increased body weight and a higher predisposition to fat deposition, as well as the altered expression of genes involved in lipid metabolism and elevated levels of reactive oxygen species and proinflammatory metabolites in adipose tissue [50]. This pattern of high postnatal body weight and increased fat deposition has been reported by other authors in mice offspring produced after in vitro embryo culture and IVF [29, 48]. These alterations are associated with accelerated neonatal growth (i.e., catch-up growth). In line with results from human studies, animal models have indicated that the alterations caused by ART depend on the specific procedure used (ICSI, IVF, or BB), and are more severe or become evident at later stages of life. Feuer et al. reported that IVF mouse offspring until 17 weeks of age presented a phenotype comparable to that of naturally conceived offspring; however, after this time, female offspring presented increased body weight, fat deposition, B cell dysfunction, and hyperinsulinemia [50]. Studies using rodent models have suggested that some metabolic or other alterations in ART-produced offspring present only later in postnatal life, usually when the offspring reaches adulthood [51].

Several researchers have studied the molecular mechanisms behind the observed adverse offspring outcomes. Rianudo et al. demonstrated that IVF mouse offspring had an altered liver and serum metabolomic profile, with the main changes corresponding to the pathways responsible for glucose metabolism [53]. Zheng et al. reported that the liver of mouse ART offspring had decreased activity of enzymes involved in glucose metabolism and that the insulin signaling pathway was altered [49], which could explain the altered glucose homeostasis. Another study reported that ART affects liver development, which results in altered lipid metabolism and the potential to develop fatty liver [54]. Overall, animal studies not only corroborate what has been observed in human studies but also provide evidence of lifelong observations of the alterations caused by ART to the cardiovascular system, adipose tissue, and liver, which are the main axes involved in the development of several metabolic diseases, including metabolic syndrome.

## Epigenetics and ART

It has been well established that adverse environmental factors can induce permanent changes in the metabolic signaling pathways of fetal tissues, causing nonreversible effects on fetal growth and development that lead to the development of metabolic diseases in adult offspring (reviewed in [55, 56]). This concept is known as fetal metabolic programming. Even though the exact mechanism behind the onset of metabolic disease is still not clear, it has been postulated to be regulated by epigenetic alterations.

Epigenetics refers to the mechanisms that modify and regulate gene expression that do not involve changes in the DNA sequence [57]. Such mechanisms include chromatin structure and modifications, DNA methylation of cytosine bases, and histone protein modifications [58]. As a consequence, epigenetic modifications are responsible for phenotypic plasticity in the absence of genetic variability, which can be influenced by environmental factors. For example, maternal under- or over-nutrition is known to induce epigenetic modifications that affect gene expression in the pathways associated with a range of physiologic processes (e.g., abnormal fetal growth, energy balance regulation, adipocyte differentiation/maturity, and hepatic metabolism). These modifications lead to adverse metabolic phenotypes during adult life: obesity, insulin resistance, and cardiovascular risk, among others [59–61]. One of the most studied epigenetic mechanisms is DNA methylation, which is known to play a critical role in the genomic imprinting (parental-specific expression by silencing one allele) that occurs during gametogenesis; genomic imprinting is maintained throughout life and can affect long-term metabolic disease susceptibility [62, 63]. Several studies have reported that alterations in methylation of specific genes lead to increased risk of developing metabolic disease [56]. For instance, Xie et al. reported that IUGR leads to an insulin-resistant phenotype by altering the DNA methylation and transcriptional activity of PGC-1α [60]. Moreover, epigenetics and imprinted genes play a critical role in the development and function of the placenta. Deletions or disruption of imprinted genes in mice show an altered balance of fetal and placental growth, evidenced by low efficiency of the placental nutrient supply [64, 65], which may lead to fetal growth restriction and thus constitutes a risk for metabolic alterations in later life. In our previous study on sheep, expression of demethyltransferase was reduced in the early placenta from in vitro-produced embryos, which is associated with growth arrest and subsequent death of the embryos [66]. Moreover, aberrant methylation of imprinted genes such as IGF2 has been associated in human placenta with fetal growth retardation, indicative of poor nutrient supply [67]. Thus, any alteration can affect growth morphology and nutrient transfer, which can impact embryonic growth and development, leading to the adult onset of metabolic disease [68, 69]. Evidence of alterations in DNA methylation patterns associated with the development of several metabolic diseases has been described in human and animal studies. Epigenetic modifications of adiponectin and leptin genes, caused by a high-fat maternal diet, cause a metabolic syndrome-like phenotype in adult mice offspring [70]. Moreover, in obese mice, hypermethylation in the promoter region of adiponectin has been associated with an exacerbation of metabolic disease symptoms [59]. In particular, one of the most consistently observed epigenetic defects involves alterations in methylation of the imprinted regions H19/IGF2 [56, 71–73]. This imprinted gene plays key roles in placental nutrient transport, size, and morphology [74]. Changes in H19/IGF2 methylation patterns have been associated with IUGR and the subsequent development of obesity, T2D, and metabolic syndrome (reviewed in [56, 75].

The genome undergoes several phases of epigenetic programming during development [76, 77]. Two main critical windows of epigenetic programming have been well described in murine models: (i) gametogenesis, where germinal cells undergo DNA remethylation of the genome, including imprinted regions; and (ii) early embryo development, which is characterized by global demethylation (with the exception of imprinted genes) [78, 79]. During these periods of epigenetic programming, the genome is particularly susceptible to environmentally induced epigenetic defects. The many procedures of ART coincide with these critical windows of epigenetic programming, thus it is likely that perturbations of the environment associated with ART could lead to stable changes in the epigenome, resulting in adverse outcomes during later life.

Epidemiological studies in the past years have gathered information about imprinting disorders and epigenetic alterations in the cord blood and fetal and placental tissues of ART-conceived children. Several authors have reported, in different birth cohorts, differential methylation patterns not only in imprinted genes involved in placental development and nutrition [80–82] but also in genes involved in adipocyte differentiation and insulin signaling [82]. Most of these studies have found decreased methylation of the H19/IGF2 and SNRPN locus, associated with upregulation of gene expression [81, 83] in the placentas of IVF/ICSI pregnancies compared to naturally conceived ones [80, 83–85]. This result could explain the IUGR found in ART-conceived children and related metabolic alterations.

Some studies have suggested that ART procedures are also able to induce changes in methylation patterns in nonimprinted regions of the genome [82, 86, 87]. Moreover, this differential methylation occurs not only in the placenta but also in fetal tissues, and the changes appear to be tissue-specific [82, 85, 88, 89]. However, an important question—are these alterations due to the ART procedures themselves or do they reflect the underlying infertility of the parents?—remains difficult to answer due to confounding factors in human studies. Thus, researchers have turned to the use of animal models, in particular rodent models, to study the effects of ART procedures on epigenetic and imprinting alterations.

Animal studies support the findings in human studies: embryo culture and ART procedures (in the absence of infertility) can alter the epigenome and cause placental and fetal abnormalities. Studies in in vitro-produced sheep and cows exhibit abnormal placental and fetal development with alterations on the epigenetic control of imprinted genes, which often result in increased pregnancy loss and perinatal mortality [18, 66, 90, 91]. Hiendleder et al. reported that IVF in cows affects methylation levels in fetal tissue, in particular, hypermethylation of the fetal liver, that was found to be associated with fetal overgrowth [92] A previous study from our group reported that embryonic growth arrest is associated with impaired placental expression and activity of DNA methyltransferase in in vitro-produced sheep embryos, which then results in hypomethylation and silencing of imprinted genes [66]. Similar findings in porcine embryos show that DNA methylation patterns were adversely affected by in vitro embryo production [93, 94]. Several authors have reported that different conditions of embryo culture in mouse can cause alterations in methylation and expression of imprinted genes involved in the growth and development of the embryo and placenta (*H19, Snrpn, Grb10*) [95–98]. A similar pattern of demethylation has been observed in the H19 locus with superovulation [99]. Furthermore, Li et al. showed that ART leads to decreased methylation levels at *H19*, *KvDMR1*, and *Snrpn* in the placenta, as well as altered expression of paternally imprinted genes involved in fetal growth [100]. Other authors have reported that IVF can alter methylation of genes involved in lipid metabolism in the placenta and can cause downregulation of these genes [30].

Altered epigenetic programming has been observed also in offspring somatic tissues (e.g., liver, brain) besides placenta and at different stages of life. Zheng et al. found differentially methylated genes related to liver development and associated with glucose (e.g., *Chrebp*, which has been associated with fatty liver and glucose intolerance) and insulin metabolism (e.g., *TNFa*, which can reduce insulin signaling) in 7.5 days postcoitum embryos [49]. These altered methylation patterns can influence abnormal glucose metabolism and liver-derived insulin resistance in adulthood. Epigenetic alterations can also influence the development of alterations during adulthood. Rexhaj et al. reported that adult ART mice offspring present an altered methylation pattern of genes responsible for vascular processes, which could explain the vascular dysfunction found in these mice [44].

**Figure 3:**
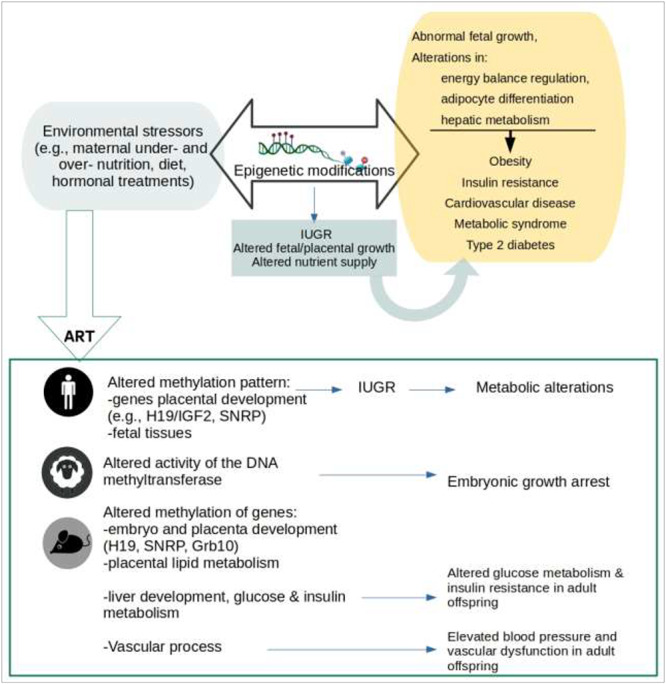
Epigenetics and ART. Adverse environmental factors can influence development through epigenetic modifications. Epigenetic modifications are associated with altered placental development and nutrient transport, as well as intrauterine growth restriction (IUGR), which can all lead to the onset of adult metabolic diseases. There is evidence from human and animal studies that ART can induce stable epigenetic modifications that result in adverse outcomes later in life.

Together, these studies provide evidence that ART can alter epigenetic reprogramming, which affects fetal growth and metabolism and could result in the development of metabolic alterations during adulthood (Figure 3). Considering the high stability of epigenetic modifications, it is possible that the alterations in the methylation patterns at early developmental stages persist later in life. However, there is also evidence that epigenetic changes are reversible and can be modulated by the environment during critical windows of susceptibility. In the case of ART, one study showed that epigenetic alterations found in blood during the neonatal period were attenuated in adulthood [101], suggesting that the adverse health outcomes associated with ART might not be permanent.

### Transgenerational inheritance of disease

In the last decade, transgenerational epigenetics has been considered a critical underlying mechanism of transgenerational inheritance of environmentally influenced disease [102–104]. This phenomenon refers to the transmission of a specific phenotype to the next generation of offspring via epigenetic modifications in the germline [105]. Transgenerational epigenetics has been reported to be involved in the transmission of metabolic disturbances, such as insulin resistance, obesity, and metabolic disease, among others, to second and third generations, in studies on maternal/paternal nutritional effects [103, 106, 107]. Furthermore, it was reported that methylation alterations in the promoter region of H19 were present in two subsequent generations in an IUGR rat model [75].

This mechanism of inheritance is an emerging topic in ART since a great number of ART-conceived children have now reached marriageable age, and the possibility of transgenerational inheritance of the adverse outcomes associated with ART should be taken into serious consideration. A few studies have addressed this subject in animal models. Calle et al. observed the transmission of metabolic abnormalities in two generations of mouse offspring generated by suboptimal embryo culture [52]. Li et al. found that differentially methylated regions of the brain in IVF mouse offspring persisted to a second generation [108]. Moreover, Xu et al. reported that alterations in the methylation pattern of several genes in the testis of adult mice were conserved in a second generation [109]. These studies reveal the need for more detailed analyses of ART-induced epigenetic alterations that could lead to the development of pathologies in adult life, not only for the health of the individual in question but also for that of future generations.

## Phenotypical plasticity

The extent and severity of adverse metabolic outcomes associated with ART are still controversial, as not all IVF/ICSI children present the same degree of metabolic alterations. It is evident that ART procedures affect metabolic health and disease in a sex-specific manner. Female ART-conceived offspring are more susceptible to cardiovascular alterations and obesity at a young age than their counterpart male offspring [42, 46]. However, in male offspring, the alterations become more evident later in life, suggesting the possible risk of further disease development [36, 42]. Sexual dimorphism is common in several metabolic alterations during adulthood (e.g., glucose metabolism and adipose tissue function), which can influence disease susceptibility [110]. Due to transcriptional sexual dimorphism, male and female genomes react differently to environmental stress, leading to sex-specific, long-term effects, and epigenetic alterations [111]. However, the difference between a mild or more severe phenotype could be explained by developmental plasticity. This phenomenon of the same environmental stressor leading to different phenotypes was observed in a rat model of in utero androgen excess, where female offspring presented two distinctive phenotypes, both with alterations in reproductive and metabolic function. However, one phenotype presented more severe alterations and was less receptive to pharmacological treatment [112, 113]. Until recently, the long-term outcomes of developmental programming were considered to be irreversible; however, there is evidence of the reversibility of the postnatal programmed phenotype. In particular, some authors have observed improvements in some components of metabolic syndrome and cardiometabolic alterations with nutritional and exercise therapy [114, 115] during critical windows of susceptibility [116]. Thus, some of the milder adverse metabolic alterations found in ART-conceived children might be reversible with proper therapeutic interventions.

## Conclusions and closing remarks

Research into the potential side effects of ART on metabolic health still has a long way to go. There is a serious need for long-term and transgenerational studies in ART-conceived children. In the paragraphs above, we have described evidence of signs of cardiometabolic alterations that are detectable at a young age, but do not appear to constitute a high risk of disease and morbidity. However, this possibility should be taken into consideration since CVD, T2D, and metabolic syndrome are chronic, adult-onset diseases, and the presence of at least one minor metabolic alteration can result in a high risk of disease later in life. Animal models have corroborated this theory, as several metabolic alterations and risk of adult disease have been reported. Studies have highlighted that at an advanced age, the alterations seem to be more severe and that a stressful environment has the ability to worsen them. We have also gathered evidence that ART is able to induce epigenetic changes that could be responsible for adverse health outcomes, and these epigenetic alterations can be transmitted across generations, culminating in the transmission of metabolic disease. However, there is evidence that the risk factors for metabolic syndrome could be reversed by changes in lifestyle (e.g., nutritional therapy, diet restrictions, physical activity), and whereas lifestyle interventions may be able to improve long-term health, the adverse outcomes may be difficult to overcome if developmental plasticity is no longer present. Thus, detection of metabolic and epigenetic alterations during the early years of ART-conceived children, as well as the proper planning of and counseling regarding therapeutic interventions, could help to prevent the development of adult-onset disease.

**Conflict of interest**: The authors have declared that no conflict of interest exists.
